# Liquid Biopsy’s Role in Head and Neck Tumors: Changing Paradigms in the Era of Precision Medicine

**DOI:** 10.3390/diagnostics15172262

**Published:** 2025-09-07

**Authors:** Rudy N. Zalzal, Najla Fakhruddin, Rami Mahfouz

**Affiliations:** 1 Faculty of Medicine, American University of Beirut, Beirut 1107 2020, Lebanon; rnz04@mail.aub.edu; 2 Department of Pathology and Laboratory Medicine, American University of Beirut Medical Center, Beirut 1107 2020, Lebanon; nf62@aub.edu.lb

**Keywords:** liquid, biopsy, head, neck, tumors, impact

## Abstract

In the era of precision medicine, liquid biopsy has become an indispensable tool in research and clinical diagnostics. This innovative molecular approach offers significant benefits in managing cancers, such as melanoma, colorectal cancer, lung cancer, and, now, head and neck tumors. By addressing the challenges posed by tumor heterogeneity, liquid biopsy aligns seamlessly with precision medicine strategies, providing more accessible methods to detect and monitor genetic alterations in tumors. While biomarkers for head and neck tumors have been identified, integrating these markers into diagnostic workflows remains a challenge due to the complexities of current sampling techniques. Could liquid biopsy pave the way for a breakthrough in the diagnosis, monitoring, and treatment of patients with head and neck cancer? This review explores the expanding role of liquid biopsy in oncology, with a focus on its potential to revolutionize the management of head and neck malignancies. Liquid biopsy has become an integral part of diagnosis, classification, and prognostication of numerous cancers, namely, head and neck tumors. Liquid biopsy should work in concert with histological testing, imaging, surgery, and other diagnostic and therapeutic approaches, as each offers distinct advantages that together contribute to achieving the best possible patient outcomes.

## 1. Introduction

Head and neck cancers (HNCs) rank as the sixth most common malignancy globally, accounting for approximately 4.5% of all cancer diagnoses around the world and 450,000 deaths annually [1]. Head and neck tumors can develop in or around the oral cavity, pharynx, larynx, nose, sinuses, and salivary glands [2]. HNCs are a heterogeneous group of malignancies, with squamous cell carcinoma (HNSCC) arising from the epithelial or mucosal surface being the predominant subtype (approximately 90%). Less common histological variants include adenocarcinomas of the salivary glands, mesenchymal tumors (such as sarcomas), and lymphomas—most notably those originating in the nasopharynx. In clinical practice, molecular profiling of tumors has become crucial for better approaching the diagnosis, prognosis, and treatment of HNSCC—especially now during the era of exome- and genome-wide sequencing [3]. At the present time, tissue biopsy (such as needle biopsy) is still considered as the gold standard for head and neck tumor examination [4]. Nonetheless, tissue biopsy faces many limitations (Table 1), including sampling error, inaccessibility, poor detection of heterogeneous and metastatic tumors, limited repeatability, and invasiveness [5,6]. This is where liquid biopsy, also known as fluid biopsy, showcases its potential (Table 1) in overcoming these shortcomings: It has the potential of demonstrating the presence of all the existing subclones and interpreting tumor cells in blood samples, far from the primary site where the tumor initially developed [7]. Moreover, liquid biopsy’s ability to pinpoint malignancy in peripheral blood accurately traces and assesses the progression of disease within the patient’s body by performing the procedure at different instances [8]. Because tumors are dynamic and constantly evolving, it is essential to consider their spatial and temporal characteristics to fully understand tumor heterogeneity and progression. Liquid biopsy’s low costs, time, and noninvasive nature have further bolstered the medical community’s interest in using liquid biopsies to detect cancer-specific biomarkers over the past ten years [9]. It refers to the analysis of tumor-derived material released into easily accessible bodily fluids, providing a noninvasive approach for cancer diagnosis, prognosis, and therapeutic monitoring. The main components of liquid biopsy include circulating tumor cells (CTCs), circulating tumor DNA (ctDNA), extracellular vesicles (EVs), and tumor-derived metabolites. Due to limitations with screening and physical examination, HNSCC commonly presents in advanced disease states associated with poor survival rates, where approximately 10% of cases have already developed distant metastasis at diagnosis [10]. In such a scenario, liquid biopsies may significantly contribute toward early malignancy detection in HNSCC patients [11]. This review aims to explore the emerging role of liquid biopsy in head and neck tumors, examining its clinical applications, advantages over traditional tissue-based methods, current evidence, and future directions in the context of precision oncology.

## 2. Head and Neck Tumors

HNSCC is divided into two categories, HPV-negative and HPV-positive HNSCC as follows:

### 2.1. HPV-Negative HNSCC

Tobacco, which contains dozens of carcinogenic chemicals, is the primary risk factor for the development of HPV-negative HNSCC [12]. These chemicals include polycyclic aromatic hydrocarbons (PAHs), nitrosamines, arsenic, and many others. Tobacco-derived carcinogens are excreted via detoxification enzymes and pathways; however, many of their reactive metabolites form covalent DNA adducts, which may lead to mutations if not repaired [13]. This outcome depends on the balance between metabolic activation from one end and detoxification plus DNA repair from another. Tobacco products also cause inflammation in the exposed tissues, producing pro-angiogenesis and proliferation cytokines, chemokines, and growth factors that ultimately promote carcinogenesis. Inflammatory cytokines, such as Interleukin-6 (IL-6) and Tumor Necrosis Factor-alpha (TNF-α), together with immune-cell-recruiting chemokines, such as CXCL8 and CCL2, are upregulated in response to tobacco-related oxidative stress, promoting chronic inflammation and a tumor-supportive microenvironment [14]. This is enhanced by Vascular Endothelial Growth Factor (VEGF), which promotes new blood vessel formation, and Epidermal Growth Factor (EGF), which stimulates epithelial cell proliferation. Alcohol overuse is another key risk factor for HPV-negative HNSCC [15]. It is metabolized into acetaldehyde, a well-known DNA adductor. In addition, alcohol serves as a solvent for many carcinogens, which exposes epithelial cells to more substances. The progression to invasive HNSCC follows a series of steps beginning with epithelial cell hyperplasia, followed by low-grade dysplasia, high-grade dysplasia, carcinoma in situ, and eventually resulting in invasive carcinoma. HPV-negative HNSCC is largely driven by mutations in tumor suppressor genes (TSGs) [13]. The progression of normal head and neck epithelial mucosa to hyperplasia occurs due to the loss of the 9p21 region, which includes the TSGs: *CDKN2A* (encoding the CDK4 and CDK6 inhibitor p16INK4A) and *ARF* (encoding p14, a stabilizer of p53). Progression to dysplasia is characterized by the loss of TP53 at the 3p21 and 17p13 regions. The transition to carcinoma in situ is marked by the loss of 11q13, 13q21, and 14q32, and the progression to invasive carcinoma involves the loss of 6p, 8, 4q27, and 10q23. *CDKN2A* and *TP53* are the two most frequently altered TSGs in HNSCC tumors, with mutations accounting for 22% for *CDKN2A* and 72% for *TP53* [13]. In addition, approximately 20% of head and neck malignancies were attributed to mutations in the oncogene *PIK3CA*. These oncogenic transformation events give rise to cancer stem cells (CSCs) that are capable of self-renewal and pluripotency.

### 2.2. HPV-Positive HNSCC

**Figure 1:**
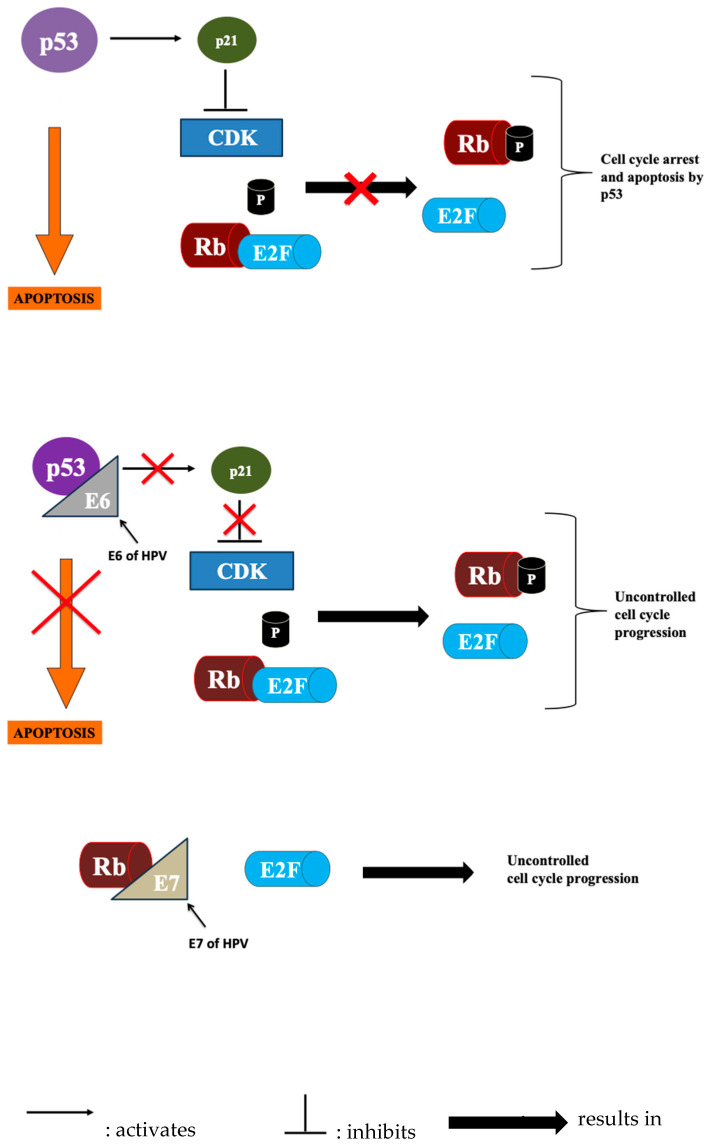
Illustration of various genes and their roles in cell cycle progression and arrest.

Increasingly, oropharyngeal cancers are associated with infection with oncogenic strains of human papillomavirus (HPV), mainly HPV-16 and HPV-18 (HPV-31, HPV-33, and HPV-52 are also present in a subset of patients) [16]. HPV is a relatively small, naked, icosahedral virus with a circular double-strand DNA genome and is the most common sexually transmitted disease (STD) worldwide. HPV’s genome consists of seven early genes (E1-E7), responsible for viral genome replication and transcription, and two late genes (L1-L2), encoding viral capsid proteins. HNSCC tumor specimens typically reveal viral genomes integrated at different sites within the host genome. The process leading to the host cell’s malignant transformation includes the activation of E6 and E7, which oncogenic activities stem from the enhanced degradation of p53 and Rb, respectively [17]. These oncoproteins (E6 and E7) disrupt normal cell cycle regulation and cause genome instability via an intricate protein–protein interaction [18]. In its non-phosphorylated state, Rb is bound to E2F, a family of transcription factors, keeping it inactive and preventing entry beyond the G1-S restriction point. The cell cycle is allowed to progress when E2F is liberated, in turn, driving the expressions of genes needed for DNA synthesis (like cyclins and DNA polymerases). This liberation of E2F is achieved when the Rb-E2F complex is disrupted and the viral proteins E6 and E7 target this pathway. E7 inactivates Rb directly, releasing E2F, while E6 acts on the p53/p21 pathway. Normally, p53 induces cyclin-dependent kinase inhibitor, p21, which inhibits CDK activity. When CDK is inhibited under the effect of p53, Rb is in its non-phosphorylated state, bound to E2F, hence preventing cell cycle progression. When E6 destroys p53, the induction of p21 is suppressed, CDK activity is left unchecked, and Rb is phosphorylated, so E2F is constitutively released. This prevents apoptosis and cell cycle checkpoint activation when DNA damage occurs, leading to uncontrolled DNA replication and genomic instability, ultimately contributing to the onset of cancer (Figure 1). In virus-associated cancers, the viral DNA (vDNA) is clonally integrated into the tumor genome, making it a highly specific and sensitive biomarker for liquid biopsy. Because viral DNA is foreign, its presence in circulating cell-free DNA (ctDNA) is a near-specific indicator of tumor presence, as it is not found in healthy cells. Viral ctDNA assays are among the most clinically ready in HNCs because they are tumor-specific, have already been evaluated in large clinical trials, and serve as models for how other ctDNA biomarkers may be clinically translated (check Section 5.2 for more details).

## 3. Treatment

Treatment of early-stage HNSCC (stages I and II) is via a single modality, like surgery or radiotherapy, while treatment of more advanced stages (III and IV) requires a combination of surgery and radiotherapy, with or without chemotherapy, depending on stage and pathological features [19]. For cases that are not curable, palliative chemotherapy is the mainstay of the treatment [20]. These modalities of treatment are devised by the multidisciplinary team after a histopathological analysis is made, the tumor extent is assessed, and detailed insight using the latest imaging platforms (MRI, CT, PET) is acquired. Because the advancement of the cancer stage necessitates more aggressive treatment, which greatly increases morbidity, and because upscaling the treatment does not guarantee a cure, earlier diagnosis remains the most desirable means of increasing the likelihood of a cure, especially in metastatic HNCs [21]. The implementation of a liquid biopsy through serial blood samples has the potential to detect metastasis earlier, therefore improving the selection of treatment plans and ameliorating the prognosis. The typical therapies for head and neck tumors, such as chemotherapy and radiotherapy, increase the patient’s overall survival (OS) rate and provide effective outcomes in the short run. In the long run, however, they lead to adverse effects on patient welfare because of the continuous exposure to radiation and chemotherapeutic drugs [22]. Instead, emerging molecular diagnostic tools and biomarker discovery have paved the way for targeting specific tumorigenic sites within the concerned region, with fewer adverse effects on neighboring tissues. Moreover, the increasing prevalence of resistance to chemotherapy and radiotherapy adds to the necessity of exploring more effective and tolerable targeted tools to ameliorate the clinical outcomes of HNC patients [23]. In brief, targeted treatments aim to develop inhibitors against specific biomarkers that are analyzed using liquid biopsy to optimize therapy for the diagnosed tumor [24], albeit improvements in molecular diagnostics, such as cetuximab, a monoclonal antibody targeting the epidermal growth factor receptor (EGFR), remains the only Food and Drug Administration (FDA)-approved EGFR-targeted therapy in HNSCC [23]. The current clinical focus has turned to immunotherapy with antibodies targeting T-cell inhibitory receptors that function as immune checkpoints [25]. The immune system plays a major role in tumor progression, and immune evasion by tumors contributes to the development of carcinogenesis. Hence, stimulating an anti-tumor immune response has been put into practice with the advent of immune checkpoint inhibitors (ICIs) that target the immune checkpoint receptors. These receptors include cytotoxic T-lymphocyte-associated protein 4 (CTLA-4), programmed cell death protein 1 (PD-1), and programmed death-ligand 1 (PD-L1) and normally function to regulate immune responses to microbes and prevent autoimmunity. Tumor cells might take advantage of the immune-checkpoint-mediated inhibition of immune responses, resulting in cancer growth. Hence, ICIs exert their action by lifting this suppression off, in turn, allowing the T-cells to kill tumor cells.

### 3.1. Anti-PD-1-Axis-Targeted Therapy

PD-1, programmed death-1, is expressed on antigen-activated T-cells and recognizes two ligands, called PD-L1 and PD-L2, expressed mainly on antigen-presenting cells and tumor cells [26]. The interaction of PD-L1 on cancer cells or APCs with PD-1 will promote immune escape by suppressing metabolic reprogramming in T-cells, reduced effector T-cells as well as memory T-cells, and shattered T-cell profusion. In a study investigating PD-L1 expression levels as a diagnostic marker for HNSCC prognosis, the results showed significantly higher expression in metastatic HNSCC. Two PD-1 inhibitors, pembrolizumab and nivolumab, have gained FDA approval for use in recurrent/metastatic HNSCC after progression through platinum-based chemotherapy. Interestingly, anti-PD-1-targeted therapies show more significant responses in patients positive for both PD-L1 and PD-L2 (27.5%) as compared to those positive for PD-L1 only (11.4%), suggesting that PD-1 interaction with both PD-L1 and PD-L2 is predictive of anti-PD-1-axis-targeted therapy responses. PD-L1 overexpression at the end of the treatment is an important independent prognostic factor linked to shorter progression-free survival (PFS), less overall survival (OS), and worse outcomes compared with those of PD-L1-negative counterparts [27].

### 3.2. Anti-CTLA-4 

CTLA-4 expressed on the activated T-cells or Tregs inhibits T-cell activation by costimulatory CD28 by competitively binding B7 on APCs. Anti-CTLA-4 monoclonal antibodies block the ability of CTLA-4 from binding to B7 molecules, thus allowing CD28 to continuously bind to B7 and activate T-cells [27]. Normally, CTLA-4 aims to minimize healthy tissue damage by stimulating immune response effectively; however, cancer cells promote their expression by secreting TGF-β, resulting in T-cell exhaustion and immune evasion. Interestingly, intracellular and surface CTLA-4 expression in CD8+ T-cells is higher in laryngeal squamous cell carcinoma patients compared to control subjects. In addition, oral squamous cell carcinoma (OSCC) patients who exhibit a low presence of CTLA-4+ cells at the invasive front of the tumor have been shown to have higher recurrence-free and metastasis-free survival rates [28]. At the present time, there are no approved CTLA-4-targeting treatments for HNSCC. Nevertheless, trials underway are testing the effectiveness of combination therapies, including anti-CTLA-4 antibodies alongside other ICIs and/or chemotherapy and radiotherapy.

While chemoradiotherapy remains the standard treatment modality for locally advanced head and neck squamous cell carcinoma, in combination with surgery or stand-alone, immune checkpoint inhibitors (ICIs) and chemotherapy are now central to the management of recurrent or metastatic disease. However, the treatment response is highly variable, underscoring the need for noninvasive biomarkers to guide patient selection, monitor therapeutic efficacy, and detect resistance. Liquid biopsy represents a promising tool to address these challenges.

## 4. Liquid Biopsy

Liquid biopsy refers to obtaining patient bodily fluid samples, such as blood, saliva, urine, or cerebrospinal fluid, for cancer diagnosis and prognosis [24]. In the context of head and neck malignancies, liquid biopsy has proven feasibility in detecting different biomarkers, such as circulating tumor cells (CTCs), cell-free tumor DNA (ctDNA), proteins, metabolites, and extracellular vehicles (EVs). Through these biomarkers, liquid biopsy has demonstrated its ability to serve as a valuable diagnostic and prognostic tool [29]. Liquid biopsy has been studied in HPV-negative squamous cell carcinoma of the head and neck (SCCHN), HPV-related SCCHN, oncogene-driven salivary gland cancers, and EBV-positive nasopharyngeal cancer [30]. With precision medicine increasingly setting the foundation of many medical practices, strategies that detect and monitor tumor genetic changes could be adopted to develop a genetic profile that forms the basis of selecting targeted and precise therapies for head and neck tumors [31]. Although liquid biopsy examinations are rarely performed in regular clinical practice, massive and phenomenal advancements have been made recently and will continue to surface in hope of maximizing patient outcomes using this promising technique.

## 5. Liquid Biopsy Biomarkers in HNSCC

Reaching a credible and comprehensive background of tumor genetics and molecular foundations requires analyzing a combination of macromolecules (Table 2).

### 5.1. Circulating Tumor Cells (CTCs) 

CTCs are cancer cells originating from primary tumor sites and capable of entering the vasculature to disseminate to distant sites of the body [3]. These transient cancer cells can be isolated from surrounding blood cells via a label-dependent method that selects CTCs based on markers expressed on their surfaces, such as epithelial cell adhesion molecules (EpCAMs) and cytokeratins (CKs), or via a label-independent method that isolates CTCs based on differences in physical properties, such as density and size [3]. Because the expression of such markers is downregulated during CTC epithelial–mesenchymal transition (EMT), such techniques (which are collectively called positive-selection methods) may fail to capture EMT-transformed CTCs. Negative-selection methods targeting cell markers on RBCs, such as CD45 and CD235a, which are not expressed on CTCs, have addressed this issue, albeit not perfectly. After CD45 leukocytes are removed, many non-tumor cells that are negative for this marker, such as endothelial cells, debris, and apoptotic and progenitor cells, persist in blood, leading to false positives. Despite these limitations, CTC detection can be reliable and clinically useful when approached with multi-modal strategies. Label-independent methods complement immunoaffinity-based techniques by depending on physical property differences. Size-based microfiltration captures CTCs, which are generally larger (15–25 µm) than blood cells (~8 µm), and density gradient centrifugation separates cells based on their density using a medium (usually Ficoll-Paque or Histopaque). After isolating CTCs, confirmatory post-isolation steps are critical to ensure that captured cells are truly circulating tumor cells and not contaminants. CTCs are extremely rare (approximately 1–100 CTCs/mL among billions of RBCs), making it challenging to detect and capture CTCs from whole-blood samples. New emerging platforms have been able to detect a larger CTC pool (3–133 CTCs/mL of blood), and further purification methods are projected to isolate CTCs with better efficacy [3].

The CTC count is reported to correlate with tumors’ responses to treatment, prognosis, and predictive value for metastasis in head and neck cancers. Analysis of CTC counts from 40 patients with OSCC and 47 with HNSCC undergoing concurrent chemoradiotherapy revealed that decreasing CTC levels within the first 2–4 weeks correlated with longer progression-free survival (PFS) and overall survival (OS), while persistently high CTC levels correlated with treatment resistance [32]. Worse PFS and OS were also correlated with increased baseline CTC counts in a recent meta-analysis of over 1054 HNC patients [30]. Multiple cohort studies have estimated that patients with detectable CTCs at diagnosis have a two- to threefold increase in the risks of progression or death. Baseline CTC positivity is also associated with an increased risk for recurrence. In a recent study on 119 treatment-naïve HNC patients, of 11 patients with an incomplete response at 13 weeks, 10 had detectable baseline CTCs [33]. This means that CTC counts are predictors of outcomes at several time points throughout a patient’s treatment: at the baseline, during chemoradiotherapy, and post surgery.

Circulating tumor cells are also useful biomarkers in metastatic HNSCC. In a retrospective cohort of 95 surgically treated HNSCC patients, CTC positivity was an independent prognostic factor in multivariate analysis and correlated with shorter progression-free survival (PFS) (*p* < 0.001) and overall survival (OS) (*p* = 0.001) [34]. This correlation between the tumor burden in circulation and survival outcomes is dose dependent, where patients with >2 CTCs/mL had a median OS of 22 months, compared to 40 months for those with ≤2 CTCs [35]. Higher CTC counts were associated with a worse prognosis, with an area under the receiver-operator-characteristic (ROC) curve of 0.756. The N stage and clinical stage were significantly associated with CTC positivity in patients with HNSCC (*p* < 0.05), with more CTC counts detected in late N and clinical stages (*p* < 0.001) [34]. Another meta-analysis of seventeen studies involving more than one thousand patients confirmed that CTC-positive HNSCC patients had more advanced TNM stages (III–IV) and a significantly higher likelihood of lymph node metastases [36]. In addition to individual CTCs, a pilot study assessing 60 HNC patients suggests an equally important clinical utility for CTC clusters—defined as ≥3 tumor cells, held in close proximity by strong cell–cell adhesions—detected in the blood of cancer patients [37]. While individual CTCs were detected in 33% of the patients, 25% had CTC clusters. Interestingly, all the cluster-positive patients had stage IV disease, which significantly correlates cluster presence with the development of distant metastasis [36]. The increased metastatic capacity of clusters compared to individual CTCs could be attributed to mechanical stability in the bloodstream, provided by the cell–cell adhesion; immune evasion due to the presence of platelets, fibroblasts, and other immune cells that create a shield around the tumor cells; increased stemness properties compared to those of individual cells; etc. [38]. Although the current landscape of CTC-based studies in HNSCC has mainly utilized blood/plasma/serum-derived samples, the presence of CTCs has been detected in saliva [39]. The feasibility of CTCs in salivary detection remains uncertain because of the shedding of normal epithelial cells along with cancerous cells in saliva and the limited numbers, which make isolation and detection difficult. Although CTCs are rare in saliva compared to blood, studies have demonstrated the promising potential of circulatory CTCs for diagnosis, prognosis, and therapeutic monitoring in HNSCC, especially in oral squamous cell carcinoma (OSCC) patients. Higher CTC counts detected using microfluidic and immunomagnetic separation methods were found to correlate with advanced-TNM-stage and lymph node metastasis. CTCs can predict the risk of metastasis in HNC patients, even before clinical examination, serving as useful tools in risk prediction.

CTCs are dynamic biomarkers, reflecting not only tumor burden but also subclone-specific biology. CTC interpretation helps in making therapy-type decisions by predicting patients’ responses to certain therapeutic drugs through uncovering the underlying biological molecules associated with the disease. Such biomarkers give valuable insight into the nature of genes and proteins implicated in a tumor. A recent study investigated four HNSCC patients, comparing their in situ tumors to matched CTCs using single-cell RNA sequencing (scRNA-Seq) [40]. CTCs showed high variability in gene expression and mutation profiles within and between patients, with subclonal diversity in CTCs matching those found in tumors. This suggests that treatment resistance may develop at the single-cell level, potentially explaining therapy failure in early-stage HNSCC. The investigators identified novel CTC biomarkers, which may enable early detection of relapse and prediction of metastasis before being detectable by imaging. Some of the dysregulated pathways carried by CTCs include CREB signaling (an oncogenic driver in HPV-positive HNSCC), β-adrenergic signaling (which promotes proliferation, invasion, and angiogenesis), and G-protein receptor signaling. A separate study identified 238 tumor-related SNVs across 120 commonly mutated genes in primary tumors, lymph nodes, and bone marrow [41]. Many mutations were unique to each site, indicating spatial heterogeneity, and distinct subclones of tumor cells in lymph nodes were identified at various histopathological stages, indicating temporal monitoring. When future studies scale up patient numbers to validate utility in routine clinical practice, these biomarkers can be translated into low-cost PCR-screening assays that will revolutionize precision oncology.

Apart from CTCs’ premise as a prognostic biomarker, these cells have a role in modulating disease amid the promising rise of immunotherapy. Recent studies have highlighted that PD-L1 is frequently expressed on CTCs and may be involved in immune evasion [37]. Programmed death 1 (PD1) checkpoint inhibitors may block the PD-1/PD-L1 immune checkpoint pathway on CTCs and activate the immune system to eliminate them, thus helping in disease remission [3]. Liquid biopsy is capable of assessing PD-L1 overexpression in CTCs and can be used for treatment response monitoring in HNSCC patients on PD1 inhibitors. CTCs have been approved by the U.S. Food and Drug Administration (FDA) as surrogate markers in breast, colorectal, and lung cancer but not in HNSCC [42]. Although data emphasize CTCs’ diagnostic and prognostic relevance in HNSCC, further clinical trials are necessary before CTCs become established diagnostic and prognostic tools in managing HNSCC patients.

### 5.2. Cell-Free Tumor DNA (ctDNA)

When a tumor grows, it releases DNA into bodily fluids, such as plasma and saliva, allowing tumor-forming genetic alterations in bodily fluids to act as diagnostic and predictive biomarkers in HNSCC patients [11]. This was evidenced when a multiplex PCR analysis of 93 saliva DNA samples from HNSCC patients showed tumor DNA in 100% of the oral cavity squamous cell carcinoma (OCSSC) patients and 50–70% of the oropharyngeal and laryngeal cancer patients [11]. Tumor DNA was also found in over 80% of the matched plasma samples, suggesting high detection frequency of tumor DNA from the oral cavity as well as different histological sites. Tumor-specific alterations, such as mutations, methylation, microsatellite instability, and gene rearrangements, distinguish ctDNA from normal cell-free DNA, making ctDNA a useful biomarker [43].

Increased ctDNA concentrations in cancer patients, which are also indicative of severity, form the basis of considering ctDNA as a diagnostic and prognostic tool. One study including 200 patients with HNSCC showed higher levels of ctDNA compared to healthy patients, and another study on 359 patients across different types of cancer (including HNSCC) correlated increased ctDNA quantity with more advanced metastatic stages [3]. The presence of ctDNA after treatment correlated with significantly worse progression-free survival (PFS) and overall survival (OS). In addition, ctDNA can be helpful as a marker of recurrence and disease progression in real time, as demonstrated by a recent study of 20 cases of HNSCC treated with radical therapy [44]. Seven cases had recurrence, while the rest did not relapse. Among the recurrent cases, ctDNA was detected in five plasma samples during follow-up, even predicting recurrence before imaging, while in the non-relapsed group, ctDNA was undetectable in all thirteen cases. Furthermore, ctDNA in pre-treatment plasma was found in 10 of 20 patients, and the detection frequency tended to increase as the disease stage progressed. Further, ctDNA’s ability to capture temporal heterogeneity was shown in a longitudinal cohort of 41 HNSCC patients treated with surgery ± adjuvant therapy [45]. Serial ctDNA sampling at surgery, 19 weeks post-op, and 6 months post-op detected less ctDNA load over time. Additionally, ctDNA detects clonal evolution and the emergence of resistance mutations throughout treatment, which were initially not present in tumor biopsies [45].

Notably, ctDNA levels are also tightly correlated with gross tumor volume such that higher volumes of the primary tumors and their involved lymph nodes were associated with higher ctDNA levels [46]. In addition to the correlation between ctDNA levels and gross tumor volume, an association between ctDNA detection and metabolic tumor burden, determined with FDG-PET/CT, in HNSCC has been observed recently [47]. This relationship is transforming how we approach HNSCC management, pushing toward an integrated model where blood-based liquid biopsies and functional imaging are used synergistically to guide more precise and personalized patient care. A recent study that enrolled twenty-six HNSCC patients undergoing FDG-PET/CT and venous liquid biopsy sampling revealed that ctDNA detection using an NGS-based commercial panel for genomic analysis is a promising tool for the genomic profiling of head and neck squamous cell carcinoma. A positive liquid biopsy, defined as a maximum variant allele frequency (VAF) in blood of ≥1.00% or ≥5.00%, correlated positively with whole-body metabolic tumor volume (MTV), as captured by and derived from an FDG-PET/CT scan [47]. While a traditional CT scan measures physical size but cannot distinguish between the active tumor, necrotic tissue, or fibrosis, MTV quantifies the total volume of biologically active, glucose-avid tumor tissue. The fact that ctDNA levels correlate with MTV makes ctDNA an equally powerful, and potentially more dynamic, prognostic liquid biomarker to be used alongside staging. Further, ctDNA provides real-time, functional information about treatment efficacy, complementing the “snapshot” provided by a single PET/CT scan. The combination of MTV and ctDNA may provide the most robust prognostic model. A patient with both a high MTV and a high level of ctDNA would be at the very highest risk of treatment failure and distant metastasis, while a patient with a low MTV and undetectable ctDNA would have an excellent prognosis [48]. This combined model could be used to stratify patients for treatment intensification or de-escalation trials. A PET/CT scan can provide a comprehensive picture of the total active disease burden, offering a highly accurate baseline risk stratification, but PET/CT cannot be safely or practically repeated weekly, and PET/CT may show equivocal uptake post treatment due to inflammation from the radiation mucositis rather than the cancer itself. Hence, ctDNA levels allow for “real-time” adaptation of therapy because they are easily and safely measured frequently [49]. The presence of ctDNA would strongly suggest active residual disease, while its absence would support a benign inflammatory process. Upon a confirmed rise in ctDNA, a PET/CT can be performed to precisely locate the site of the recurrence, often before it is visibly large on a CT scan.

Further, ctDNA reflects mutations from various parts of the tumor, even those not reached by a traditional biopsy, allowing clinicians to capture the full genetic complexity of a tumor [50]. A pilot cohort of nine treatment-naïve HNSCC patients extracted DNA from two intra-tumoral sites (core and margin) as well as from blood, then analyzed all the samples using a nine-gene mutation panel [51]. A total of 86.9% of the COSMIC (Cancer Mutation Database) driver mutations were found in only one tumor region, meaning that most key mutations were localized and not spread throughout the whole tumor. The percentage of genes localized to only one portion of the tumor was significantly less for ctDNA (56%) because of the various subclones across the tumor shedding DNA into the circulation.

Recent analysis of ctDNA sequencing found that patients with head and neck cancer had the highest number of ctDNA alterations detected (88%) among many different tumor types, such as gastrointestinal, brain, breast, and lung, speaking to the potential utility of ctDNA sequencing in HNSCC [52]. While ctDNA was detected in 81% of the patients with active disease, the sensitivity was higher in metastatic disease (91%) than in locally advanced tumors (72%) [53]. This means ctDNA could help to monitor if cancer is spreading, without invasive biopsies. Finding certain mutations (like TP53 or BRCA2) in blood can predict if a patient’s cancer will spread or respond poorly to treatment. HPV-positive tumors have significantly fewer TP53 and DNA repair alterations per patient, pointing toward better survival [53]. The TP53 gene, so critical that it is considered as the “guardian of the genome”, is the most frequently altered gene (in this study, it was found in 51% of the ctDNA) and is associated with a three- to fourfold increased risk of death [53]. Mutations in other genes, such as CDKN2A, TERT (which often co-occurs with TP53 mutations), and BRCA2 (important for prognostic purposes), are associated with poorer outcomes and shorter survival. This is important because 65.3% of the patients had actionable ctDNA alterations that can guide clinical decision making and treatment [53]. FDA-approved PARP and PI3K inhibitors may be used off-label for the treatment of BRCA1/2- and PIK3CA-mutated HNSCC. Some alterations in DNA repair genes, including ATM/APC, may also respond to chemotherapy or immunotherapy [53]. At the present time, cetuximab (anti-EGFR) remains the only FDA-approved treatment for HNSCC, highlighting unmet needs. These findings were elucidated from a pivotal study exploring the genomic landscape of head and neck squamous cell carcinoma from two DNA sources: circulation and tumor tissue [53]. Tumor DNA (tDNA) isolated from biopsies/surgeries and circulating DNA from processed blood samples were each sequenced and then detected mutations were compared in tDNA vs. ctDNA. It showed that only 13% of the mutations overlapped between the blood and tissue, with highest concordance rate in TP53, underlining the importance of blood tests in complementing tissue-biopsy-guided treatment.

Besides mutations, epigenetic mechanisms of gene expression could be used as biomarkers in bodily fluids for early disease detection, as changes in gene expression can be identified long before the phenotype is manifested [11]. The feasibility of the methylation analysis of plasma-circulating cell-free DNA was supported by ctDNA from 6689 patients with over 50 cancer types, including HNSCC. Whole-genome bisulfite sequencing and a panel targeting over 100,000 distinct methylation regions localized the tissue of origin for many cancer types at metastatic and non-metastatic stages with >90% accuracy. Methylation analyses can be conducted not only on blood-based assays but also on saliva. A recent study using methylation-specific PCR to assess the promoter methylation status of several tumor-suppressor genes in DNA from OCSCC and OPSCC patients showed significant hypermethylation in these genes compared to those in normal controls [11], which is expected because inducing transcriptional silencing or downregulation of TSGs contributes to carcinogenesis. Although tumor-associated mitochondrial DNA mutations (mtDNA) and copy number changes in bodily fluids of patients with HNSCC have not yet been extensively studied, mtDNA alterations have also been linked to risk, progression, and treatment responses of HNSCC. Because mtDNA has a high copy number and more stability than genomic DNA due to its circular nature, it can serve as a powerful molecular marker for HNSCC detection in biopsies, surgical margins, and lymph nodes. Due to its short half-life in blood circulation, this biomarker is also highly valuable in examining dynamic driver mutations and tumor load changes over time.

Lastly, ctDNA is especially useful in virus-associated tumors, namely, HPV-driven oropharyngeal cancer and EBV-driven nasopharyngeal cancer [54]. Unlike somatic mutations in human DNA, which require deep sequencing to be distinguished from normal background signals, viral DNA is entirely foreign to the human genome. This fundamental feature explains the significant advancement of viral ctDNA as biomarkers for viral cancers over non-viral etiology. Evaluated techniques ready for clinical use include more routine methods, like quantitative PCR (qPCR) and droplet digital PCR (ddPCR), which amplify and quantify a specific, short viral DNA sequence, and more advanced modalities, such as next-generation sequencing (NGS), which profile target regions along the entire viral genome with selected human genes in parallel. The application for HPV-driven oropharyngeal cancer (HPV-OPC) is rapidly evolving and highly promising. Pre-treatment HPV ctDNA levels correlate with disease burden and prognosis, with increased pre-treatment HPV ctDNA being linked to more advanced nodal disease [55]. Interestingly, viral ctDNA could also be used to monitor responses and the minimal residual disease (MRD), where clearance of HPV ctDNA predicted a cure, while persistence predicted recurrence. A pivotal study following 115 patients with serial ddPCR found that patients with undetectable HPV ctDNA at the end of radiotherapy had an exceptional 2-year recurrence-free survival (RFS) of 95% while that in patients with detectable ctDNA was only 65% [55]. The test identified 100% of the recurrences with a median lead time of 3.9 months before clinical recurrence. This confirms the high sensitivity and specify of ctDNA as viral load markers with minimal risk of false results. Adding to how this biomarker is being integrated into prospective clinical decision making, another ongoing trial (NRG-HN005) aims to use ctDNA clearance for guiding therapy de-escalation and reducing drug toxicity [56]. Undetectable HPV ctDNA at week 2 or 4 of radiotherapy is used to randomize patients into reduced-dose radiotherapy groups to determine if de-intensified radiation therapy regimens are non-inferior to standard-of-care chemoradiation.

Another application of viral ctDNA is EBV-driven nasopharyngeal carcinoma (NPC). Plasma EBV DNA can detect preclinical, early-stage NPC, playing important roles in screening and early diagnosis. A landmark study screening 20,174 asymptomatic men in Hong Kong found EBV DNA to be a powerful tool for population-based screening in endemic areas, with a sensitivity of 97.1, a specificity of 98.6%, and a positive predictive value of 11.0%. Pre-treatment an EBV DNA load is also a strong prognostic factor and tool for monitoring treatment responses such that a high baseline EBV DNA was found to be associated with worse survival outcomes: twofold higher risks of death, recurrence, or metastasis. Post-treatment EBV DNA was also strongly correlated with an eightfold higher risk of distant metastasis [57]. The unique biology of virus-associated cancers has propelled applications like EBV DNA for NPC to the forefront of liquid biopsy and is rapidly doing the same for HPV-OPC.

### 5.3. Extracellular Vehicles (EVs)

EVs are released into the tumor microenvironment (TME) by cancer cells for intercellular communication purposes that promote cell growth and survival [58]. They are divided into three subgroups, depending on their cell of origin, diameter, and surface protein markers: exosomes (40–100 nm), micro-vesicles (50–1000 nm), and apoptotic bodies (50–2000 nm). EVs contain an array of bioactive cargo, from proteins and mRNA transcripts to miRNAs and long non-coding RNAs. The multiple functions of extracellular vesicles in head and neck cancer carcinogenesis and development include facilitating proliferation and invasion, regulating tumor immunology, remodeling the tumor microenvironment, and promoting angiogenesis [58].

Exosomal miRNA collected from HNSCC patients’ saliva differs substantially from miRNA sequencing profiles seen in controls, providing insight into exosomes as potential liquid biopsy biomarkers. Studies have shown that saliva EVs from oral cancer patients are larger in size, more irregular in morphology, aggregate more readily, and, interestingly, have unique infrared signatures compared with those of EVs from controls [59]. A systematic review across nine original studies identifies forty-one differentially expressed miRNAs in HNSCC saliva versus that of healthy controls, with miR-10b-5p, miR-486-5p, miR-24-3p, miR-412-3p, and miR-512-3p being the most promising for early detection, while miR-1307-5p, miR-21, and miR-519c-3p correlated with worse survival outcomes [60]. Exosomal miR-21 downregulates tumor suppressors PTEN and PDCD4 and is higher in resistant and metastatic OSCC, making it both diagnostic and prognostic. In addition, plasma exosomal miRNA mirrors the miRNA within the HNSCC tumor tissue itself, further highlighting liquid biopsy as a tool of tumor detection when access to the primary cancer site is difficult. For instance, circulating exosomal miR-21 levels correlate with the tumor stage, lymph node metastasis, and hypoxic signaling, especially in oral squamous cell carcinoma, laryngeal squamous cell carcinoma, and esophageal squamous cell carcinoma patients [61]. Levels of most exosomal miRNAs, in patients responding to therapy, were shown to be suppressed, paving the way for circulating miRNA as a tool in HNSCC diagnosis and disease monitoring. A study comparing EV levels from the plasma of oral cancer patients before and after surgery has shown that some subpopulations of EVs were decreased [59]. Another important finding was that plasma EV levels decreased relative to pre-therapy levels in cases who responded well to treatment. In contrast, exosomal miR-196a remains elevated in cisplatin-resistant HNSCC through targeting cyclin-dependent kinase (CDK) N1B and inhibitor of growth family member 5 (ING5), indicating that this miRNA may serve as a promising predictor of cisplatin resistance and poor survival in HNSCC [62].

Aside from miRNA, long non-coding RNAs (lncRNAs) are dysregulated in a cancer-specific manner. They are non-protein-coding RNA transcripts longer than 200 nucleotides and are remarkably stable in bodily fluids, protected within exosomes or complexed with RNA-binding proteins. One of the most studied oncogenic lncRNAs across cancers, MALAT1 (Metastasis-Associated Lung Adenocarcinoma Transcript 1), was found to be significantly elevated in HNSCC patients’ plasma exosomes compared to those of healthy controls. It demonstrated high diagnostic power, with an AUC (Area Under the Curve) of 0.873, indicating excellent discriminatory ability [63]. Circulating lncRNA levels at diagnosis often predict patient outcomes such that high pre-treatment levels of circulating MALAT1 and other key lncRNAS, such as HOTAIR and ANRIL, are consistently associated with advanced TNM stages, lymph node metastasis (LNM), and poorer overall survival (OS) [63]. Serial measurement of lncRNAs offers a dynamic tool to assess treatment efficacy. For instance, patients whose exosomal MALAT1 levels did not normalize post operation had a significantly higher risk of recurrence due to residual disease [64].

Not only do EVs protect lncRNA from RNAses but also messenger RNA (mRNA) makes them preferred biomarkers over free circulating mRNA. Detection of resistance-associated mRNAs, such as Met, could predict invasive and treatment-resistant disease phenotypes, as these pathways are known drivers of poor prognosis in HNSCC. In addition, EMT transcripts within exosomes, such as EGFR variants, are enriched in advanced disease and associated with recurrence/metastasis in exploratory cohorts, as the epithelial-to-mesenchymal transition enhances motility, invasiveness, and resistance to apoptosis [65].

Also, screening the protein signature of the EV cargo from patients’ plasma using antibody arrays underlines EVs’ potential roles in prognosis and response assessment. Exosomes in the plasma of patients with HNCs carry immunosuppressive molecules and interfere with functions of immune cells [66]. Exosome-induced immune suppression correlates with disease activity in HNCs, suggesting that plasma exosomes could be useful as biomarkers of HNC progression. High levels of Programmed Death-Ligand 1 (PD-L1) on circulating EVs are strongly associated with lymph node metastasis, disease progression, and suppressed immune function in HNSCC patients [67]. Nodal metastasis and recurrence were also associated with high plasma exosomal Arginase-1 levels [68]. EVs can also be used in post-treatment surveillance because the molecular composition of the cargo differs depending on the patient’s response to the treatment. Flow cytometry showed that EXOs obtained before treatment were rich in N-Cadherin and TGF-β1 (they had a mesenchymal profile), while those collected after therapy carried more E-cadherin (they had an epithelial profile) [3]. Moreover, exosomal composition can guide therapy. When the protein cargo in plasma is rich in exosomal EGFR and phospho-EGFR, a response to cetuximab treatment is expected, for example [62]. This is because cetuximab acts as a competitive inhibitor by binding to EGFR, thereby blocking its activation by endogenous ligands [69]. EV-associated proteins are generally more reliable and specific biomarkers than free proteins in liquid biopsy for HNSCC. Although free circulating proteins are easier to detect, EV-associated proteins are generally more reliable and specific biomarkers than free proteins in liquid biopsy for HNSCC because of their higher stability and specificity. EV-associated proteins offer diagnostic, prognostic, and therapy-monitoring potentials, especially for immunotherapy markers, like PD-L1 and EMT-related proteins.

### 5.4. Metabolites

Metabolomics is an emerging discipline, and tumor metabolites have been well investigated among liquid biomarkers. It refers to the study and analysis of small-molecule metabolites (e.g., amino acids, lipids, and organic acids) found in blood, saliva, or urine to identify biomarkers for diagnosis, prognosis, treatment response, and disease monitoring. More than 50 differential metabolites have been identified in oral cancer, with 84% sensitivity, and 74% of them using capillary electrophoresis–mass spectrometry (CE-MS) [70]. For instance, stearyl alcohol and sucrose are predictive markers for OSCC patients, while plasma lysophosphatidylcholines are sensitive and cancer-specific liquid markers for squamous cell carcinomas of the esophagus [71]. A study looking into the plasma metabolites of esophageal squamous carcinoma (ESC) showed that ESC patients have abnormal fatty acid levels and elevated levels of purine metabolism byproducts (such as uric acid) compared to those of controls. Similarly, considering lipids’ key role in maintaining cell integrity, changes in the lipid profile have also been associated with cancer progression [11]. Via the use of 48 metabolites, statistical studies were able to readily discriminate between esophageal cancer patients and healthy controls, underlining the potential of metabolic products as predictive markers in head and neck tumors [72]. Furthermore, the study revealed that treatment progress could be reflected in the levels of some metabolites, as pre- and post-treatment groups were readily discriminated based on metabolic studies. To date, metabolites are the least mature of the biomarkers in HNSCC liquid biopsy, despite the growing field.

## 6. Conclusions

In the era of precision medicine, liquid biopsy is fundamentally reshaping the management paradigm of head and neck squamous cell carcinoma (HNSCC). It is transitioning patient care beyond static anatomical staging and reactive surveillance toward a dynamic molecular model. The utility of circulating tumor DNA (ctDNA), particularly from Epstein–Barr virus (EBV)- and human papillomavirus (HPV)-driven cancers, is demonstrating unprecedented value in early detection, refining prognoses independent of the clinical stage and providing real-time monitoring of treatment responses. Most significantly, it offers a predictive lead time for detecting recurrence, enabling proactive and potentially curative salvage therapy. Crucially, the emerging predictive role of liquid biopsy must be emphasized. While currently most impactful in virus-associated HNSCC, the ongoing discovery of biomarkers and actionable mutations promises to guide future targeted therapies, personalizing treatment based on the real-time genetic landscape of a patient’s disease. This shift does not undermine the irreplaceable roles of tissue diagnosis, histopathology, or imaging. Instead, liquid biopsy serves as a complementary pillar, integrating molecular data with conventional modalities to create a holistic view of the disease. The current limitations (Table 3 and Table 4) are challenges of implementation not of principle. Future efforts must focus on validating standardized protocols and exploring novel bio-sources, like saliva, particularly for oral cavity cancers. Ultimately, the integration of liquid biopsy into multidisciplinary care is the cornerstone of next-generation oncology. By providing a continuous stream of molecular data, it empowers clinicians to make more informed, timely, and precise decisions. This upgrades HNSCC management from a one-size-fits-all approach to a truly dynamic and personalized strategy aimed at maximizing survival and minimizing morbidity.

## Figures and Tables

**Table 1:** Clinical limitations of tissue biopsy and corresponding advantages of liquid biopsy in head and neck squamous cell carcinoma.

Disadvantages of Tissue Biopsy	Advantages of Liquid Biopsy
Sampling error: May fail to capture the most representative part of the tumor	Offers a more comprehensive molecular profile by capturing circulating tumor DNA (ctDNA) from multiple tumor sites
Limited to localized and easily accessible tumors, with difficulty in identifying metastasis	Powerful in detecting tumors distant from primary site via analysis of bodily fluids (e.g., blood)
Poor detection of tumoral heterogeneity	Potential to exhibit the presence of all the existing subclones
Pain and discomfort caused by its invasive nature	Minimally invasive and easy-to-collect nature
Non-real-time monitoring: incapable of performing the procedure consecutively on patients due to invasive nature	Repeatability and serial sample collection over time allows for evaluation and assessment of disease progression
Costly and time-consuming	Low cost and time required for collecting samples

**Table 2:** Advantages and disadvantages of different liquid biopsy biomarkers.

	Advantages	Disadvantages
**CTCs**	Provides a complete and necessary overview (genomics and proteomics); Enables testing on living cells, both in vitro and in vivo; Presence of immune checkpoint markers on CTCs allows for immunotherapeutic studies	Few found in circulation and in other fluids; Contamination with healthy neighboring cells, such as leukocytes; Requires rapid processing of fresh blood samples; Loss of epithelial markers (Ep-CAM-negative CTCs) during EMT and misses mesenchymal-type CTCs; Low sensitivity in early-stage and HPV+ disease; Some methods (e.g., filtration and centrifugation) can damage fragile CTCs, preventing downstream culture or functional assays
**CtDNA**	Isolated from different sources and many different biofluids; Less biofluid volume is necessary (when compared to CTCs); Associated with many cancer types; Allows for the detection of many genetic mutations; Its short half-life allows for tracking of tumor load changes	A small amount of material is shed in the circulation, especially in early-stage and locally advanced HNCs; Release of DNA from normal cells dying from apoptosis or other processes may interfere with tumor ctDNA detection
**EVs**	Provide comprehensive information from many biomolecules (DNA/RNA/proteins); Present in large amounts in several bodily fluids; miRNA in EV cargo is relatively stable and not easily degraded and, hence, readily stored	Lack of standardization for detection and isolation; Difficulty in specifically isolating tumor-derived EVs from the total EV population; Biological properties must be validated: EV size and content
**Metabolites**	Specific signatures (such as high lactic acid and ketone body levels) are correlated with tumor burden, cancer stage, and treatment resistance in pilot studies. Help to understand the tumor microenvironment (e.g., hypoxia and Warburg effect)	Low tumor specificity and confounded by systemic factors; Require complex computational models to identify meaningful patterns; Large prospective validation studies are lacking Pre-analytical stability is a major concern

**Table 3:** Problems of liquid biopsy implementation in the routine laboratory.

Disadvantages of Liquid Biopsy
Detection of both ctDNA and CTC is not achievable to the same extent at all tumoral sites within the same patient, even within the same tumoral entity.
Large bodily fluid samples are required
Optimal sensitivities and specificities of different biomarkers have not yet been definitively established. ****
Standardization gap: No universal protocol across institutions:
- Sample collection: Plasma vs. serum, different anticoagulants (EDTA vs. citrate vs. Streck tubes); - Sample processing: Time of centrifugation, centrifugation speed, number of spins, and storage conditions can all significantly impact the yield and quality; - Lack of universally accepted standard operating procedures (SOPs) leads to inter-laboratory variability, making it difficult to compare results across different hospitals or studies; - Lack of standardized cut-off thresholds: There are no universally agreed upon thresholds for what constitutes a “positive” result for prognosis or residual disease.
Anatomical and biological considerations:
- The primary tumor’s location can influence biomarker release such that tumors in well-vascularized areas might shed more than those in more confined or necrotic areas, making liquid biopsy an unreliable tool for those specific patients; - Low abundance of analytes: The ctDNA may represent <0.1% of the total ctDNA; CTCs may be 1–10 cells per 10 mL of blood.
Operational barriers:
- Major oncology guidelines (e.g., NCCN and ESMO) have not yet incorporated liquid biopsy as a standard-of-care recommendation for most HNC scenarios outside of clinical trials, halting reimbursement and widespread adoption; - Health insurance providers often consider liquid biopsy for HNCs as “investigational” or “not medically necessary” due to the lack of guideline recommendations; - Implementing a robust liquid biopsy program is especially challenging for low- and middle-income countries. It requires specialized equipment (like digital PCR and specialized microfluidic platforms), skilled personnel with a high level of expertise in molecular diagnostics, and bioinformatics pipelines for interpretation.

**** Comparative overview of CTCs, ctDNA, EVs, and metabolites as liquid biopsy biomarkers in head and neck cancers, with emphasis on sensitivity, specificity, predictive value, and translational potential.

**Table 4:** Evaluating CTCs, ctDNA, EVs, and metabolites for diagnostic and translational potential.

Parameter	CTCs	ctDNA	EVs	Metabolites
**Sensitivity**	Moderate: ~40–70%	High (with NGS/ddPCR): ~75–95% in metastatic HNSCC, ~50–70% in localized disease	Sensitivity for tumor-derived EVs is not yet well quantified because although plasma contains billions of EVs/mL, the tumor-specific signal is diluted by the background from normal cells.	Moderate–High: Sensitive platforms can detect pM-nM concentrations. Specific tumor-derived metabolic signatures are still being defined.
**Specificity**	High: Relies on positive selection for epithelial (EpCAM) and/or tumor-specific markers and negative selection for CD45 (leukocytes).	Very high for mutation-specific assays (e.g., detecting a known TP53 mutation or HPV-DNA)	Moderate: Challenging to distinguish tumor-derived EVs from host EVs. Specificity depends on identifying unique surface markers or cargo (e.g., EGFRvIII and HPV-RNA).	Low–Moderate: Metabolic changes are highly influenced by non-tumor factors (diet, inflammation, exercise, and comorbidities).
**Key Clinical Applications**	- Prognostic stratification - Pharmacodynamic biomarker for therapy monitoring - Functional studies (e.g., cultures and xenografts) to study metastasis	- “Molecular Residual Disease” (MRD) detection - Real-time therapy response monitoring - Identifying actionable/targetable genomic alterations - Early relapse detection	- Early detection and diagnosis - Monitoring immune checkpoint expression (such as PD-L1+ EVs) - Intercellular communication research - Potential as drug delivery vehicles	- Identification of metabolic therapeutic targets - Adjunct to imaging for diagnosis and monitoring - Understanding the tumor microenvironment (hypoxia and Warburg effect)
**Translational Readiness Level for HNSCC**	Intermediate: An FDA-cleared platform (CellSearch^®^) exists but is primarily prognostic. Not yet the standard of care	High (Rapidly Advancing): ctDNA for MRD and genotyping is entering clinical guidelines via NCCN for other cancers; HNSCC-specific validation is ongoing	Low–Intermediate (Preclinical/Early Clinical): Active area of research. No standardized clinical assays exist yet	Low (Preclinical): Primarily in the research domain; not yet ready for clinical decision making

ddPCR: droplet digital PCR; NGS: next-generation sequencing.

## Data Availability

No new data were created or analyzed in this study.

## References

[1] Miserocchi G., Spadazzi C., Calpona S., De Rosa F., Usai A., De Vita A., Liverani C., Cocchi C., Vanni S., Calabrese C. (2022). Precision Medicine in Head and Neck Cancers: Genomic and Preclinical Approaches. J. Pers. Med..

[2] Son E., Panwar A., Mosher C.H., Lydiatt D. (2018). Cancers of the Major Salivary Gland. J. Oncol. Pract..

[3] Kong L., Birkeland A.C. (2021). Liquid Biopsies in Head and Neck Cancer: Current State and Future Challenges. Cancers.

[4] Oliveira K.C.S., Ramos I.B., Silva J.M.C., Barra W.F., Riggins G.J., Palande V., Pinho C.T., Frenkel-Morgenstern M., Santos S.E.B., Assumpcao P.P. (2020). Current Perspectives on Circulating Tumor DNA, Precision Medicine, and Personalized Clinical Management of Cancer. Mol. Cancer Res..

[5] Mader S., Pantel K. (2017). Liquid Biopsy: Current Status and Future Perspectives. Oncol. Res. Treat..

[6] Siravegna G., Marsoni S., Siena S., Bardelli A. (2017). Integrating liquid biopsies into the management of cancer. Nat. Rev. Clin. Oncol..

[7] Baccelli I., Schneeweiss A., Riethdorf S., Stenzinger A., Schillert A., Vogel V., Klein C., Saini M., Bäuerle T., Wallwiener M. (2013). Identification of a population of blood circulating tumor cells from breast cancer patients that initiates metastasis in a xenograft assay. Nat. Biotechnol..

[8] Rodríguez J., Avila J., Rolfo C., Ruíz-Patiño A., Russo A., Ricaurte L., Ordóñez-Reyes C., Arrieta O., Zatarain-Barrón Z.L., Recondo G. (2021). When Tissue is an Issue the Liquid Biopsy is Nonissue: A Review. Oncol. Ther..

[9] Arantes L., De Carvalho A.C., Melendez M.E., Lopes Carvalho A. (2018). Serum, plasma and saliva biomarkers for head and neck cancer. Expert Rev. Mol. Diagn..

[10] Hanna G.J., Lau C.J., Mahmood U., Supplee J.G., Mogili A.R., Haddad R.I., Jänne P.A., Paweletz C.P. (2019). Salivary HPV DNA informs locoregional disease status in advanced HPV-associated oropharyngeal cancer. Oral Oncol..

[11] Mishra V., Singh A., Chen X., Rosenberg A.J., Pearson A.T., Zhavoronkov A., Savage P.A., Lingen M.W., Agrawal N., Izumchenko E. (2022). Application of liquid biopsy as multi-functional biomarkers in head and neck cancer. Br. J. Cancer.

[12] Barsouk A., Aluru J.S., Rawla P., Saginala K., Barsouk A. (2023). Epidemiology, Risk Factors, and Prevention of Head and Neck Squamous Cell Carcinoma. Med. Sci..

[13] Johnson D.E., Burtness B., Leemans C.R., Lui V.W.Y., Bauman J.E., Grandis J.R. (2020). Head and neck squamous cell carcinoma. Nat. Rev. Dis. Primers.

[14] Wang M., Xiao Y., Miao J., Zhang X., Liu M., Zhu L., Liu H., Shen X., Wang J., Xie B. (2025). Oxidative Stress and Inflammation: Drivers of Tumorigenesis and Therapeutic Opportunities. Antioxidants.

[15] Di Credico G., Polesel J., Dal Maso L., Pauli F., Torelli N., Luce D., Radoï L., Matsuo K., Serraino D., Brennan P. (2020). Alcohol drinking and head and neck cancer risk: The joint effect of intensity and duration. Br. J. Cancer.

[16] Sabatini M.E., Chiocca S. (2020). Human papillomavirus as a driver of head and neck cancers. Br. J. Cancer.

[17] Diana G., Corica C. (2021). Human Papilloma Virus vaccine and prevention of head and neck cancer, what is the current evidence?. Oral Oncol..

[18] Jain M., Yadav D., Jarouliya U., Chavda V., Yadav A.K., Chaurasia B., Song M. (2023). Epidemiology, Molecular Pathogenesis, Immuno-Pathogenesis, Immune Escape Mechanisms and Vaccine Evaluation for HPV-Associated Carcinogenesis. Pathogens.

[19] Riva G., Pecorari G. (2021). Multimodality and Sequential Therapy in Locally Advanced Head and Neck Cancer: A Preface to the Special Issue. Cancers.

[20] Barham W.T., Stagg M.P., Mualla R., DiLeo M., Kansara S. (2025). Recurrent and Metastatic Head and Neck Cancer: Mechanisms of Treatment Failure, Treatment Paradigms, and New Horizons. Cancers.

[21] Grisanti S., Almici C., Consoli F., Buglione M., Verardi R., Bolzoni-Villaret A., Bianchetti A., Ciccarese C., Mangoni M., Ferrari L. (2014). Circulating tumor cells in patients with recurrent or metastatic head and neck carcinoma: Prognostic and predictive significance. PLoS ONE.

[22] Taylor K.J., Amdal C.D., Bjordal K., Astrup G.L., Herlofson B.B., Duprez F., Gama R.R., Jacinto A., Hammerlid E., Scricciolo M. (2023). Serious Long-Term Effects of Head and Neck Cancer from the Survivors’ Point of View. Healthcare.

[23] Li Q., Tie Y., Alu A., Ma X., Shi H. (2023). Targeted therapy for head and neck cancer: Signaling pathways and clinical studies. Signal Transduct. Target. Ther..

[24] Temilola D.O., Adeola H.A., Grobbelaar J., Chetty M. (2023). Liquid Biopsy in Head and Neck Cancer: Its Present State and Future Role in Africa. Cells.

[25] Qian J.M., Schoenfeld J.D. (2020). Radiotherapy and Immunotherapy for Head and Neck Cancer: Current Evidence and Challenges. Front. Oncol..

[26] Wongpanuwich W., Yodsanga S., Chaisuparat R., Amornphimoltham P. (2022). Association Between PD-L1 and Histatin1, 3 Expression in Advanced Head and Neck Squamous Cell Carcinoma. Anticancer Res..

[27] Mestiri S., El-Ella D.M.A., Fernandes Q., Bedhiafi T., Almoghrabi S., Akbar S., Inchakalody V., Assami L., Anwar S., Uddin S. (2024). The dynamic role of immune checkpoint molecules in diagnosis, prognosis, and treatment of head and neck cancers. Biomed. Pharmacother..

[28] Koike K., Dehari H., Ogi K., Shimizu S., Nishiyama K., Sonoda T., Sasaki T., Sasaya T., Tsuchihashi K., Hasegawa T. (2020). Prognostic value of FoxP3 and CTLA-4 expression in patients with oral squamous cell carcinoma. PLoS ONE.

[29] Martins I., Ribeiro I.P., Jorge J., Gonçalves A.C., Sarmento-Ribeiro A.B., Melo J.B., Carreira I.M. (2021). Liquid Biopsies: Applications for Cancer Diagnosis and Monitoring. Genes.

[30] Cabezas-Camarero S., Pérez-Segura P. (2022). Liquid Biopsy in Head and Neck Cancer: Current Evidence and Future Perspective on Squamous Cell, Salivary Gland, Paranasal Sinus and Nasopharyngeal Cancers. Cancers.

[31] Leibetseder A., Preusser M., Berghoff A.S. (2022). New Approaches with Precision Medicine in Adult Brain Tumors. Cancers.

[32] Wang H.M., Wu M.H., Chang P.H., Lin H.C., Liao C.D., Wu S.M., Hung T.M., Lin C.Y., Chang T.C., Tzu-Tsen Y. (2019). The change in circulating tumor cells before and during concurrent chemoradiotherapy is associated with survival in patients with locally advanced head and neck cancer. Head Neck.

[33] Zhang X., Weeramange C.E., Hughes B.G.M., Vasani S., Liu Z.Y., Warkiani M., Hartel G., Ladwa R., Thiery J.P., Kenny L. (2024). Circulating tumour cells predict recurrences and survival in head and neck squamous cell carcinoma patients. Cell. Mol. Life Sci..

[34] Zhou S., Wang L., Zhang W., Liu F., Zhang Y., Jiang B., Wang J., Yuan H. (2021). Circulating Tumor Cells Correlate With Prognosis in Head and Neck Squamous Cell Carcinoma. Technol. Cancer Res. Treat..

[35] Rahman M.M., Hossain M.M., Islam S., Ahmed R., Majumder M., Dey S., Kawser M., Sarkar B., Himu M.E.R., Chowdhury A.A. (2024). CTC together with Shh and Nrf2 are prospective diagnostic markers for HNSCC. BMC Mol. Cell Biol..

[36] Sun T., Zou K., Yuan Z., Yang C., Lin X., Xiong B. (2017). Clinicopathological and prognostic significance of circulating tumor cells in patients with head and neck cancer: A meta-analysis. Onco Targets Ther..

[37] Jansson S., Bendahl P.-O., Larsson A.-M., Aaltonen K.E., Rydén L. (2016). Prognostic impact of circulating tumor cell apoptosis and clusters in serial blood samples from patients with metastatic breast cancer in a prospective observational cohort. BMC Cancer.

[38] Kulasinghe A., Schmidt H., Perry C., Whitfield B., Kenny L., Nelson C., Warkiani M.E., Punyadeera C. (2018). A Collective Route to Head and Neck Cancer Metastasis. Sci. Rep..

[39] Patel A., Patel S., Patel P., Tanavde V. (2022). Saliva Based Liquid Biopsies in Head and Neck Cancer: How Far Are We From the Clinic?. Front. Oncol..

[40] Stucky A., Viet C.T., Aouizerat B.E., Ye Y., Doan C., Mundluru T., Sedhiazadeh P., Sinha U.K., Chen X., Zhang X. (2024). Single-Cell Molecular Profiling of Head and Neck Squamous Cell Carcinoma Reveals Five Dysregulated Signaling Pathways Associated with Circulating Tumor Cells. Cancer Control.

[41] Cao Y., Dong H., Li G., Wei H., Xie C., Tuo Y., Chen N., Yu D. (2022). Temporal and spatial characteristics of tumor evolution in a mouse model of oral squamous cell carcinoma. BMC Cancer.

[42] Kulasinghe A., Kenny L., Perry C., Thiery J.-P., Jovanovic L., Vela I., Nelson C., Punyadeera C. (2016). Impact of label-free technologies in head and neck cancer circulating tumour cells. Oncotarget.

[43] Kaorey N., Dickinson K., Agnihotram V.R., Zeitouni A., Sadeghi N., Burnier J.V. (2025). The role of ctDNA from liquid biopsy in predicting survival outcomes in HPV-negative head and neck cancer: A meta-analysis. Oral Oncol..

[44] Chikuie N., Urabe Y., Ueda T., Hamamoto T., Taruya T., Kono T., Yumii K., Takeno S. (2022). Utility of plasma circulating tumor DNA and tumor DNA profiles in head and neck squamous cell carcinoma. Sci. Rep..

[45] Marret G., Lamy C., Vacher S., Cabel L., Séné M., Ahmanache L., Courtois L., El Beaino Z., Klijanienko J., Martinat C. (2025). Deciphering molecular relapse and intra-tumor heterogeneity in non-metastatic resectable head and neck squamous cell carcinoma using circulating tumor DNA. Oral Oncol..

[46] Hilke F.J., Muyas F., Admard J., Kootz B., Nann D., Welz S., Rieß O., Zips D., Ossowski S., Schroeder C. (2020). Dynamics of cell-free tumour DNA correlate with treatment response of head and neck cancer patients receiving radiochemotherapy. Radiother. Oncol..

[47] Silvoniemi A., Laine J., Aro K., Nissi L., Bäck L., Schildt J., Hirvonen J., Hagström J., Irjala H., Aaltonen L.M. (2023). Circulating Tumor DNA in Head and Neck Squamous Cell Carcinoma: Association with Metabolic Tumor Burden Determined with FDG-PET/CT. Cancers.

[48] Jin A., Lin X., Yin X., Cui Y., Ma L. (2022). Prognostic value of MTV and TLG of 18 F-FDG PET in patients with head and neck squamous cell carcinoma: A meta-analysis. Medicine.

[49] Lele S.J., Adilbay D., Lewis E., Pang J., Asarkar A.A., Nathan C.O. (2024). ctDNA as an Adjunct to Posttreatment PET for Head and Neck Cancer Recurrence Risk Assessment. Otolaryngol. Head Neck Surg..

[50] Rapado-González Ó., Rodríguez-Ces A.M., López-López R., Suárez-Cunqueiro M.M. (2023). Liquid biopsies based on cell-free DNA as a potential biomarker in head and neck cancer. Jpn. Dent. Sci. Rev..

[51] Payne K.F.B., Brotherwood P., Suriyanarayanan H., Brooks J.M., Batis N., Beggs A.D., Gendoo D.M.A., Mehanna H., Nankivell P. (2024). Circulating tumour DNA detects somatic variants contributing to spatial and temporal intra-tumoural heterogeneity in head and neck squamous cell carcinoma. Front. Oncol..

[52] Schwaederle M., Chattopadhyay R., Kato S., Fanta P.T., Banks K.C., Choi I.S., Piccioni D.E., Ikeda S., Talasaz A., Lanman R.B. (2017). Genomic Alterations in Circulating Tumor DNA from Diverse Cancer Patients Identified by Next-Generation Sequencing. Cancer Res..

[53] Wilson H.L., D’Agostino R.B., Meegalla N., Petro R., Commander S., Topaloglu U., Zhang W., Porosnicu M. (2021). The Prognostic and Therapeutic Value of the Mutational Profile of Blood and Tumor Tissue in Head and Neck Squamous Cell Carcinoma. Oncologist.

[54] Damerla R.R., Lee N.Y., You D., Soni R., Shah R., Reyngold M., Katabi N., Wu V., McBride S.M., Tsai C.J. (2019). Detection of Early Human Papillomavirus-Associated Cancers by Liquid Biopsy. JCO Precis. Oncol..

[55] Chera B.S., Kumar S., Shen C., Amdur R., Dagan R., Green R., Goldman E., Weiss J., Grilley-Olson J., Patel S. (2020). Plasma Circulating Tumor HPV DNA for the Surveillance of Cancer Recurrence in HPV-Associated Oropharyngeal Cancer. J. Clin. Oncol..

[56] Chundury A., Kim S. (2021). Radiation Dose De-Escalation in HPV-Positive Oropharynx Cancer: When Will It Be an Acceptable Standard of Care?. J. Clin. Oncol..

[57] Alami I.E., Gihbid A., Charoute H., Khaali W., Brahim S.M., Tawfiq N., Cadi R., Belghmi K., El Mzibri M., Khyatti M. (2022). Prognostic value of Epstein-Barr virus DNA load in nasopharyngeal carcinoma: A meta-analysis. Pan Afr. Med J..

[58] Chang W.H., Cerione R.A., Antonyak M.A. (2021). Extracellular Vesicles and Their Roles in Cancer Progression. Methods Mol. Biol..

[59] Qu X., Li J.W., Chan J., Meehan K. (2020). Extracellular Vesicles in Head and Neck Cancer: A Potential New Trend in Diagnosis, Prognosis, and Treatment. Int. J. Mol. Sci..

[60] Sanesi L., Mori G., Troiano G., Ballini A., Valzano F., Dioguardi M., Muzio L.L., Magalhaes M., Caponio V.C.A. (2024). Salivary exosomal microRNA profile as biomonitoring tool for diagnosis and prognosis of patients with head and neck squamous cell carcinoma: A systematic review. Arch. Oral Biol..

[61] Hofmann L., Ludwig S., Vahl J.M., Brunner C., Hoffmann T.K., Theodoraki M.-N. (2020). The Emerging Role of Exosomes in Diagnosis, Prognosis, and Therapy in Head and Neck Cancer. Int. J. Mol. Sci..

[62] Xiao C., Song F., Zheng Y.L., Lv J., Wang Q.F., Xu N. (2019). Exosomes in Head and Neck Squamous Cell Carcinoma. Front. Oncol..

[63] Li X., Cao Y., Gong X., Li H. (2017). Long noncoding RNAs in head and neck cancer. Oncotarget.

[64] Chaudhary R., Wang X., Cao B., De La Iglesia J., Masannat J., Song F., Hernandez-Prera J.C., Gimbrone N.T., Slebos R.J., Chung C.H. (2020). Long noncoding RNA, LINC00460, as a prognostic biomarker in head and neck squamous cell carcinoma (HNSCC). Am. J. Transl. Res..

[65] Minami S., Chikazu D., Ochiya T., Yoshioka Y. (2023). Extracellular vesicle-based liquid biopsies in cancer: Future biomarkers for oral cancer. Transl. Oncol..

[66] Ludwig S., Floros T., Theodoraki M.N., Hong C.S., Jackson E.K., Lang S., Whiteside T.L. (2017). Suppression of Lymphocyte Functions by Plasma Exosomes Correlates with Disease Activity in Patients with Head and Neck Cancer. Clin. Cancer Res..

[67] Theodoraki M.N., Yerneni S.S., Hoffmann T.K., Gooding W.E., Whiteside T.L. (2018). Clinical Significance of PD-L1(+) Exosomes in Plasma of Head and Neck Cancer Patients. Clin. Cancer Res..

[68] Teng Y., Gao L., Loveless R., Rodrigo J.P., Strojan P., Willems S.M., Nathan C.-A., Mäkitie A.A., Saba N.F., Ferlito A. (2021). The Hidden Link of Exosomes to Head and Neck Cancer. Cancers.

[69] Wang L., Li L., Zhu G. (2024). Extracellular vesicle-based biomarker in head and neck cancer: Prospects and challenges. Malig. Spectr..

[70] Mohd Faizal N.F., Vincent-Chong V.K., Ramanathan A., Paterson I.C., Karen-Ng L.P., Zaini Z.M. (2024). Metabolomic Profiling of Oral Potentially Malignant Disorders and Its Clinical Values. Biomedicines.

[71] Radaic A., Kamarajan P., Cho A., Wang S., Hung G.C., Najarzadegan F., Wong D.T., Ton-That H., Wang C.Y., Kapila Y.L. (2024). Biological biomarkers of oral cancer. Periodontology 2000.

[72] Xu J., Chen Y., Zhang R., Song Y., Cao J., Bi N., Wang J., He J., Bai J., Dong L. (2013). Global and targeted metabolomics of esophageal squamous cell carcinoma discovers potential diagnostic and therapeutic biomarkers. Mol. Cell. Proteom..

